# Impact of glycemic control on biventricular function in patients with type 2 diabetes mellitus: a cardiac magnetic resonance tissue tracking study

**DOI:** 10.1186/s13244-022-01357-7

**Published:** 2023-01-11

**Authors:** Jing Zhu, Wenjia Li, Fang Chen, Zhen Xie, Kaimin Zhuo, Ruijue Huang

**Affiliations:** 1 Department of Radiology, The First Affiliated Hospital of Chengdu Medical College, Chengdu, 610041 China; 2 Department of Basic Medicine, Hainan Vocational University of Science and Technology, Haikou, 570100 China; 3 Department of Neurology, Xindu District People’s Hospital of Chengdu, Chengdu, 610041 China

**Keywords:** Type 2 diabetes mellitus, Glycemic control, Left ventricular strains, Right ventricular strains, Cardiac magnetic resonance

## Abstract

**Background:**

Poor glycemic control is associated with left ventricular (LV) dysfunction in patients with type 2 diabetes mellitus (T2DM). Nonetheless, the association between glycemic control and right ventricular (RV) function in T2DM has not been studied. This study aimed to evaluate the correlation between glycemic control and biventricular function and assess whether one ventricular function was mediated by the other ventricular changes using cardiac magnetic resonance.

**Materials and methods:**

A total of 91 T2DM patients with normal ejection fraction were enrolled and divided into two groups according to glycated hemoglobin (HbA1c) with a cut off 7%. Twenty controls were included. Biventricular ventricular strain parameters, including global peak systolic radial strain, global peak systolic circumferential strain (GCS), global peak systolic longitudinal strain (GLS), peak diastolic radial strain rate (RSR), peak diastolic circumferential strain rate (CSR) and peak diastolic longitudinal strain rate (LSR) were measured.

**Results:**

Compared with controls, patients with both HbA1c < 7% and HbA1c ≥ 7% showed significantly lower LVGCS, LVGLS, LVCSR, LVLSR, RVGLS, RVRSR, RVCSR and RVLSR. Patients with HbA1c ≥ 7% elicited significantly higher RVGCS than controls and lower LVGLS, LVCSR, LVLSR, RVGLS and RVLSR. Multivariable linear regression demonstrated that HbA1c was independently associated with LVGLS, LVLSR, RVGLS and RVLSR after adjustment for traditional risk factors. LV (RV) was not statistically mediated by the other ventricular alterations.

**Conclusion:**

In T2DM patients, glycemic control was independently associated with impaired LV and RV systolic and diastolic function and these associations were not mediated by the other ventricular changes.

**Supplementary Information:**

The online version contains supplementary material available at 10.1186/s13244-022-01357-7.

## Key points

- Biventricular strain decreased in T2DM patients with preserved EF.
- Suboptimal glycemic control aggravated the deterioration of biventricular strain in T2DM patients.
- HbA1c was independently associated with biventricular strain after adjustment for risk factors.
- Relationship of HbA1c with LV or RV strain wasn’t mediated by the other ventricular.

**Supplementary Information:**

The online version contains supplementary material available at 10.1186/s13244-022-01357-7.

## Introduction

It is estimated that there are currently 451 million people worldwide living with diabetes mellitus (DM), and it is expected to reach 650 million by 2045 [1]. Among people with DM, cardiovascular disease (CVD) is the leading cause of death, with a risk of death twice that of those without DM [2, 3]. Typically, left ventricular (LV) diastolic dysfunction is an early manifestation of DM-induced heart disease, followed by LV remodeling and systolic dysfunction, which could even lead to heart failure with the progression of DM [4–6]. Apart from the LV, several studies have proposed and confirmed that right ventricular (RV) morphology and function were also affected in DM [7, 8]. Besides, impaired RV structure and function was associated with increased risks of heart failure; RV function was shown to be a stronger predictor of mortality than LV function in heart failure with preserved ejection fraction (EF) [9]. Thus, early identification of biventricular subclinical dysfunction could facilitate earlier intervention and improves prognosis.

Cardiac magnetic resonance (CMR) imaging has emerged as the gold standard for non-invasive evaluation of biventricular structure and function in recent years [10]. The conventional measurement of ejection fraction (EF) could not evaluate myocardial regional function and is not sensitive for early myocardial systolic function impairment [10]. CMR-derived strain analysis using routinely cine sequence is an emerging modality which allows for early evaluation of global and regional subclinical myocardial dysfunction in longitudinal, circumferential, and radial directions. Myocardium consists of three layers: the inner oblique, the middle circular, and the outer oblique myocardial layers [11]. Longitudinal strain represents the longitudinal shortening of the cardiomyocytes from the base to the apex, and it is mostly determined by the longitudinally oriented myocardial fibers in the subendocardial layer [11]. Circumferential strain represents cardiomyocytes shortening along the LV circular perimeter in the short­axis, and it is mostly influenced by circumferentially oriented muscle fibers in the mid­wall [11]. Radial strain represents myocardial thickening during systole toward the center of the ventricular cavity and it is influenced by full myocardial layers [11].

Factors contributing to myocardial impairment remain to be fully investigated and identifying the underlying mechanisms could help guide decision-making. It is recognized that chronic hyperglycemia could impair mitochondrial energy signaling and cause endothelial dysfunction, resulting in intra-endothelial accumulation of advanced glycated end products and increased myocardial oxidative stress, as well as cardiomyocyte hypertrophy, collagen deposition and cross-linking, all of which may lead to myocardial remodeling and dysfunction [12–17]. Additionally, several concepts suggest that ventricular interdependence may be an additional important determinant [18–20]. Previous studies have reported that poor glycemic control is associated with subclinical LV myocardial dysfunction and remodeling in patients with type 2 DM (T2DM) [21, 22]. However, the impact of glycemic control on the RV function has not been systematically investigated. Besides, it is not clear whether the influence of glycemic control on the LV (RV) is independent of the other ventricle.

Therefore, the present study aimed to evaluate the impact of glycemic control on biventricular myocardial function in T2DM patients with preserved ejection fraction (EF) using CMR imaging strain analysis. Moreover, we explored whether the association between glycemic control and LV (RV) variations was influenced by other ventricular changes.

## Methods

### Study population

Patients with T2DM who were submitted to CMR in the First Affiliated Hospital of Chengdu Medical College from January 2019 to July 2022 (as defined by the American Diabetes Association criteria [23]) were included in the study. Patients with myocardial infarction, known coronary artery disease, clinical symptoms of heart failure or angina, significant valvular disease (moderate to severe valvular stenosis or regurgitation), Corona Virus Disease (COVID) myocarditis, bundle branch block, atrial fibrillation, chronic lung disease, pulmonary hypertension, with LV ejection fraction (LVEF) < 50% on CMR, severe hepatic (Child-Turcotte-Pugh score ≥ 10) and renal dysfunction (estimated glomerular filtration rate (eGFR) < 30 mL/min/1.73 m^2^) were excluded. Finally, a total of 91patients were enrolled. In the control group, 20 healthy individuals, age and sex-matched, with normal HbA1c and without diabetes or pre-diabetes diagnosis were enrolled. This retrospective study was in accordance with the principles of the Declaration of Helsinki and approved by the ethical committee of our institution.

### Clinical measurements

Data on demographic and clinical information were obtained based on patient reports and medical records at the time of CMR. Body mass index (BMI) was calculated as weight (kg) divided by the square of height (m). Body surface area (BSA) was computed as 0.0057 × height (cm) + 0.0121 × weight (kg) + 0.0882 for men and 0.0073 × height (cm) + 0.0127 × weight (kg)—0.2106 for women. Smoking history was defined as previous or current tobacco use. Family history of coronary artery disease (CAD) was collected. Hypertension was defined as a systolic and/or diastolic blood pressure ≥ 140 and/or 90 mmHg or the use of anti-hypertensive medication. Dyslipidemia was determined as total cholesterol (TC) > 200 mg/dl, low-density lipoprotein (LDL) ≥ 130 mg/dl and high-density lipoprotein (HDL) < 40 mg/dl for males or HDL < 50 mg/dl for females or the use of lipid-lowering medications. Fasting blood samples were acquired to measure biochemical indices such as fasting plasma glucose, glycated hemoglobin (HbA1c), TC, triglycerides, HDL and LDL. Estimated glomerular filtration rate (eGFR) was calculated based on the Modification of Diet in Renal Disease (MDRD) study equations [24]. Additionally, information regarding medications was also recorded.

### Cardiac magnetic resonance protocol

CMR examinations were performed with a 1.5 T whole-body scanner (Siemens Avanto, Erlangen, Germany) in the supine position. Electrocardiography gating (ECG) and respiratory gating technique were applied to monitor dynamic changes during the entire scanning process. Data were recorded during the breath-holding period following the end of expiration. A standard balanced steady-state free precession (bSSFP) sequence was used to obtain two-, three- and four-chamber cine images in the long-axis view, as well as a stack of continuous cine images from the apex to the base in the short-axis view. Acquisition parameters were as follows: Repetition time (TR) = 3.2 ms, echo time (TE) = 1.6 ms, flip angle = 60°, pixel size = 2.4 × 1.4 mm. To exclude perfusion defection, rest first-pass perfusion images were acquired in three standard short-axis slices (basal, middle, and apical) with dynamic inversion recovery prepared echo-planar image sequencing. The acquisition parameters were: TR/TE: 187/1.0 ms, flip angle: 50°, slice thickness: 8 mm, and matrix size: 270 × 210. To rule out myocardial infarction, late gadolinium enhancement (LGE) images were acquired with segmented-turbo-FLASH–phase-sensitive inversion recovery (PSIR) sequence 10–15 min after contrast administration. The acquisition parameters were: TR/TE: 187/1.0 ms, flip angle: 50°, slice thickness: 8 mm, and matrix size: 270 × 210. T1 mapping and T2 mapping sequence examinations were not performed in this population.

### Image analysis

CMR data were analyzed using commercial software (cvi42; Circle Cardiovascular Imaging Inc., Calgary, Alberta, Canada) by an experienced radiologist with more than three years of CMR experience. The endocardial and epicardial borders of the LV and RV myocardium on the short-axis cine images were manually traced at the end-diastolic and end-systolic phases; meanwhile, the papillary muscles and trabeculae were excluded in all series in the short-3D module. The biventricular structural and functional parameters were automatically computed, including mass, end-systolic volume (ESV), end-diastolic volume (EDV), stroke volume (SV) and EF. Mass, EDV, ESV indexed for BSA (MI, EDVI and ESVI) were calculated using the Mosteller formula [25]. Global LV and RV strain parameters analysis were performed by manually defining the endocardium and epicardium at the end-diastolic phase and automatically tracking myocardial voxel points on the short-axis, two- and four-chamber long-axis cine images. Both LV and RV strain indices included the global peak systolic radial strain (GRS), global peak systolic circumferential strain (GCS), global peak systolic longitudinal strain (GLS), peak diastolic radial strain rate (RSR), peak diastolic circumferential strain rate (CSR) and peak diastolic longitudinal strain rate (LSR).

### Reproducibility

Intra- and inter-observer reproducibility for LV and RV strain variables was measured in 30 randomly selected patients. One investigator analyzed these patients on two separate measurements with 1-month interval to evaluate for intra-observer reproducibility. For inter-observer reproducibility, a second observer blinded to the clinical and CMR results independently measured LV and RV strain parameters.

### Statistical analysis

Continuous variables are presented as the mean ± standard deviation and median (25–75% interquartile range (IQR)) for normal distribution and non-normal distribution, respectively. Categorical data are expressed as numbers (percentages). Comparisons of baseline characteristics and biventricular structure and function parameters among groups were performed using one-way analysis of variance with the Bonferroni post-hoc correction, Kruskal–Wallis rank test or Chi squared test as appropriate. Univariable and multivariable linear regression analysis was performed to assess the association of HbA1c with LV and RV function. Model 1: unadjusted regression analysis. Model 2: adjusted for age, sex, BMI, diabetic duration, smoking, hypertension, dyslipidemia, hypoglycemic mediation, FBG, eGFR and LVMI/RVMI. Mediation analyses were performed to assess whether the changes in the structure and function of one ventricle statistically mediated the association between HbA1c and the function of the other ventricle after adjustment for traditional risk factors. Thus, we added these factors (RVEF, RVMI, RVGLS, RVLSR, LVEF, LVMI, LVGLS, LVLSR) to the aforementioned linear regression models. Both independent and joint mediation effects were presented as the relative change of the regression coefficient. The corresponding 95% confidence intervals were assessed according to Preacher and Hayes (10,000 bootstrap iterations) [26]. Multicollinearity was assessed by collinearity diagnostics (i.e., tolerance < 0.1 and/or variance inflation factor > 10). Intra-class correlation coefficients (ICC) were calculated to evaluate inter-observer and intra-observer reproducibility. All analyses were performed with the statistical software package SPSS version 22.0 (SPSS IBM Corporation, Armonk, NY, USA). A two-sided *p* value < 0.05 was considered statistically significant.

## Results

### Baseline characteristics

In total, 111 individuals (20 controls and 91 patients) were included in this study. These T2DM patients were divided into two subgroups according to HbA1c levels (< 7.0% and ≥ 7.0%) based on previous studies [22]. Table 1 demonstrates the demographic characteristics and clinical data of the total study population. Participants in the three groups were comparable in age, gender distribution and BMI. Compared with controls, patients with HbA1c < 7.0% had lower eGFR values (86.96 ± 20.95 ml/min/1.732 m^2^ vs. 100.61 ± 15.45 ml/min/1.732 m^2^, *p* < 0.05) but higher FBG (6.76 ± 1.40 mmol/L vs. 5.07 ± 0.61 mmol/L, *p* < 0.05) and HbA1c (5.99 ± 0.53% vs. 5.35 ± 0.63%, *p* < 0.05), whereas patients with HbA1c ≥ 7.0% had lower eGFR (78.41 ± 24.78 ml/min/1.732 m^2^ vs. 100.61 ± 15.45 ml/min/1.732 m^2^, *p* < 0.05) but higher FBG (8.73 ± 3.31 mmol/L vs. 5.07 ± 0.61 mmol/L, *p* < 0.05) and HbA1c (7.80 ± 1.22% vs. 5.35 ± 0.63%, *p* < 0.05). As compared with T2DM patients with HbA1c < 7.0%, patients with HbA1c ≥ 7.0% exhibited significantly higher FBG (8.73 ± 3.31 mmol/L vs. 6.76 ± 1.40 mmol/L, *p* < 0.05) and HbA1c (7.80 ± 1.22% vs. 5.99 ± 0.53%, *p* < 0.05). Out of the patients with HbA1c < 7.0%, a total of 16 (29%) patients reported a smoking history, 33 (60%) patients had hypertension, and 30 (55%) patients had dyslipidemia. Meanwhile out of the patients with HbA1c ≥ 7.0%, 11 (31%) patients described a smoking history, 20 (56%) patients had hypertension and 23 (64%) patients had dyslipidemia. With regard to cardiovascular complications, 4 (7%), 5 (9%), 5 (9%) and 9 (16%) patients with HbA1c < 7.0% had retinopathy, neuropathy, nephropathy and peripheral vascular disease, respectively. Furthermore, 3 (8%) patients had retinopathy and 4 (11%) patients had neuropathy and 4 (11%) patients had nephropathy and 7 (19%) had peripheral vascular disease in the group with HbA1c ≥ 7.0%. Medications for all patients are presented in Table 1.

**Table1:** Baseline characteristics of the study cohort

	Control (*n* = 20)	T2DM patients	
		HbA1c < 7% (*n* = 55)	HbA1c ≥ 7% (*n* = 36)
Age (years)	56 ± 14	62 ± 11	58 ± 13
Male sex, *n *(%)	10 (50%)	26 (47%)	16 (44%)
BMI (kg/m^2^)	25.54 ± 2.84	27.81 ± 4.94	28.28 ± 4.32
Heart rate (bpm)	75 ± 11	83 ± 18	90 ± 29
Systolic blood pressure (mmHg)	124 ± 12	132 ± 23	128 ± 24
Diastolic blood pressure (mmHg)	72 ± 9	74 ± 14	74 ± 15
Family history of CAD, *n* (%)	2 (10%)	7 (13%)	4 (11%)
Smoking (never/former/current), *n* (%)	15/3/2 (75%/15%/10%)	39/6/10 (71%/11%/18%)	25/5/6 (69%/14%/17%)
Hypertension	0	33 (60%)	20 (56%)
Dyslipidemia	0	30 (55%)	23 (64%)
Total cholesterol (mmol/L)	3.99 ± 0.83	4.28 ± 0. 85	4.55 ± 1.34
Triglycerides (mmol/L)	1.19 (1.03, 1.99)	1.55 (0.97, 1.88)	1.57 (0.93, 1.93)
LDL (mmol/L)	2.31 ± 0.67	2.50 ± 0.70	2.64 ± 0.59
HDL (mmol/L)	1.38 ± 0.28	1.32 ± 0.35	1.25 ± 0.38
eGFR (ml/min/1.732 m^2^)	100.61 ± 15.45	86.96 ± 20.95*	78.41 ± 24.78*
FBG (mmol/L)	5.07 ± 0.61	6.76 ± 1.40*	8.73 ± 3.31*†
HbAc1 (%)	5.35 ± 0.63	5.99 ± 0.53*	7.80 ± 1.22*†
T2DM duration (years)	0	2(1.10, 3)	3 (2. 4)
*Complications*			
Retinopathy, *n* (%)	0	4 (7%)	3 (8%)
Neuropathy, *n* (%)	0	5 (9%)	4 (11%)
Nephropathy, *n* (%)	0	5 (9%)	4 (11%)
Peripheral vascular disease, *n* (%)	0	9 (16%)	7 (19%)
*Antihypertensive therapy*			
ACEI/ARB, *n* (%)	0	11 (20%)	7 (19%)
Beta-blocker, *n* (%)	0	11 (20%)	7 (19%)
Calcium channel blocker, *n* (%)	0	15 (27%)	8 (22%)
*Lipid-lowering therapy*			
Statin, *n* (%)	0	4 (7%)	4 (11%)
*Hypoglycemic therapy*			
Oral, *n* (%)	0	13 (24%)	9 (25%)
Insulin, *n* (%)	0	2 (4%)	3 (8%)

*T2DM* Type 2 diabetes mellitus, *BMI* Body mass index, *LDL* Low-density lipoprotein, *FBG* Fasting blood glucose, *HDL* High-density lipoprotein, *eGFR* Estimated Glomerular Filtration Rate, *HbA1c* glycosylated hemoglobin

*p value < 0.05 when compared with the controls group

^**†**^*p* value < 0.05 when compared with the patients with HbA1c < 7.0%

### CMR findings

Left ventricular mass index (LVMI) was higher in T2DM patients with HbA1c ≥ 7.0% than controls (61.72 ± 14.48 g/m^2^ vs. 52.30 ± 14.25 g/m^2^, *p* < 0.05). Regarding other LV functional and structural parameters, there were no significant difference among the three groups. By comparing LV strain parameters, regarding systolic function, patients with HbA1c < 7.0% had significantly lower LVGCS (− 19.05 ± 3.27% vs.  − 22.87 ± 4.11%, *p* < 0.05) and LVGLS (− 18.41 ± 3.04% vs.  − 22.89 ± 3.05%, *p* < 0.05) than controls. There were no significant differences in the LVEF and LVGRS between the patients with HbA1c < 7.0% and controls. Patients with HbA1c ≥ 7.0% had significantly lower LVGCS (− 18.27 ± 2.14% vs.  − 18.84 ± 3.29%, *p* < 0.05) and LVGLS (− 16.72 ± 3.87% vs. 18.67 ± 2.97%, *p* < 0.05) compared with controls. LVEF and LVGRS were not significantly different between the patients with HbA1c ≥ 7.0% and controls. LVGLS (− 16.72 ± 3.87% vs.  − 18.41 ± 3.04%, *p* < 0.05) was significantly lower in patients with HbA1c ≥ 7.0% than in patients with HbA1c < 7.0%. Patients in the two groups were similar in terms of LVEF, LVGRS and LVGCS. Concerning diastolic function, patients with HbA1c < 7.0% had significantly lower LVCSR (0.99 ± 0.26% vs. 1.36 ± 0.27%, *p* < 0.05) and LVLSR (1.10 ± 0.21% vs. 1.28 ± 0.26%, *p* < 0.05) than controls. No significant differences were observed in the LVRSR between the patients with HbA1c < 7.0% and controls. Patients with HbA1c ≥ 7.0% had significantly lower LVCSR (0.81 ± 0.24% vs. 1.36 ± 0.27%, *p* < 0.05) and LVLSR (0.94 ± 0.20% vs. 1.02 ± 0.22%, *p* < 0.05) compared with controls. Moreover, LVRSR values were not significantly different between patients with HbA1c ≥ 7.0% and controls. Whilst LVCSR (0.81 ± 0.24% vs. 0.99 ± 0.26%, *p* < 0.05) and LVLSR (0.94 ± 0.20% vs. 1.10 ± 0.21%, *p* < 0.05) were significantly lower in patients with HbA1c ≥ 7.0% than those with HbA1c < 7.0%. Patients in the two groups were comparable in terms of LVRSR.

**Fig. 1:**
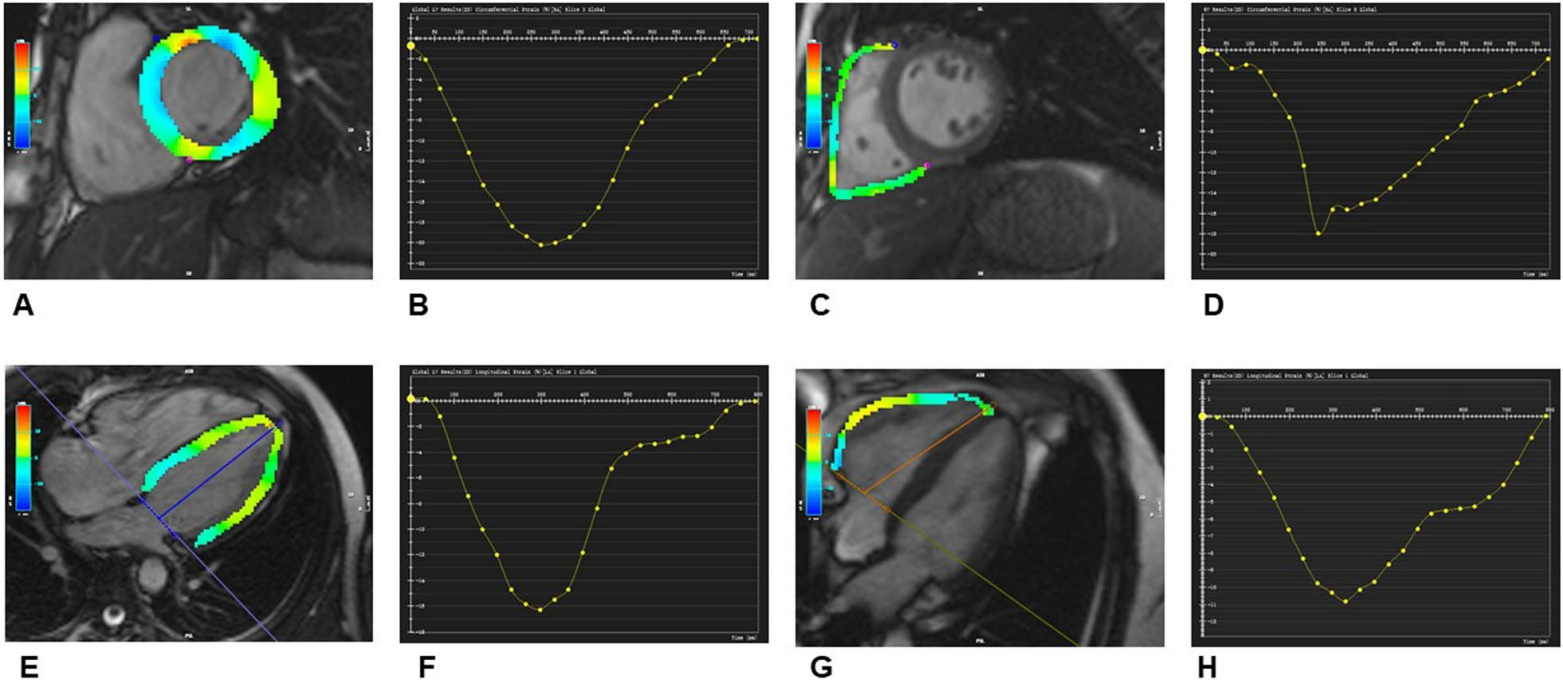
Representative CMR pseudocolor images and CMR-derived peak strain curves in T2DM patients. **A**, **C**: LV, RV pseudocolor images in short-axis; **B**, **D**: LV, RV global peak strain curve in circumferential direction; **E**, **G**: LV, RV pseudocolor images in horizontal 4-chamber long-axis; **F**, **H**: LV, RV global peak strain curves in longitudinal direction

RVEDVI, RVESVI, RVSVI and RVEF were comparable among the three groups. By comparing RV strain parameters, regarding systolic function, patients with HbA1c < 7.0% had significantly lower RVGLS (− 18.50 ± 2.32% vs.  − 21.46 ± 2.74%, *p* < 0.05) than controls. There were no significant differences in the RVEF, RVGRS and RVGCS between the patients with HbA1c < 7.0% and controls. Patients with HbA1c ≥ 7.0% had significantly lower RVGCS (− 18.60 ± 3.00% vs.  − 20.03 ± 2.79%, *p* < 0.05) and RVGLS (− 17.09 ± 3.11% vs.  − 21.46 ± 2.74%, *p* < 0.05) compared with controls. RVEF and RVGRS values were not significantly different between the patients with HbA1c ≥ 7.0% and controls. RVGLS (− 17.09 ± 3.11% vs.  − 18.50 ± 2.32%, *p* < 0.05) was significantly lower in patients with HbA1c ≥ 7.0% than patients with HbA1c < 7.0%. Patients in the two groups were similar in terms of RVEF, RVGRS and RVGCS. Regarding diastolic function, patients with HbA1c < 7.0% had significantly lower RVRSR (− 1.81 ± 0.45% vs.  − 2.65 ± 0.87%, *p* < 0.05), RVCSR (0.85 ± 0.23% vs. 1.19 ± 0.18%, *p*< 0.05) and RVLSR (0.87 ± 0.13% vs. 1.14 ± 0.18%, *p* < 0.05) than controls. Patients with HbA1c ≥ 7.0% had significantly lower RVRSR (− 1.44 ± 0.68% vs.  − 2.65 ± 0.87%, *p* < 0.05), RVCSR (0.76 ± 0.26% vs. 1.19 ± 0.18%, *p* < 0.05) and RVLSR (0.75 ± 0.14% vs. 1.14 ± 0.18%, *p* < 0.05) compared with controls. RVLSR (0.75 ± 0.14% vs. 0.87 ± 0.13%, *p* < 0.05) was significantly lower in patients with HbA1c ≥ 7.0% than patients with HbA1c < 7.0%, but RVRSR and RVCSR were not significantly different between the two groups (Table 2). Figure 1 presented representative CMR cine images and CMR-derived peak strain curves in T2DM patients.

**Table 2:** MRI-derived biventricular structure and function parameters of the study cohort

	Control (*n* = 20)	T2DM patients	
		HbA1c < 7% (*n* = 55)	HbA1c ≥ 7% (*n* = 36)
*LV structure*			
LVEDVI (mL/m^2^)	74.71 ± 12.50	73.47 ± 13.91	69.30 ± 13.64
LVESVI (mL/m^2^)	30.07 ± 7.01	30.66 ± 7.72	29.74 ± 6.14
SVI	44.65 ± 7.82	42.80 ± 8.21	39.57 ± 8.27
LVMI (g/m^2^)	52.30 ± 14.25	56.02 ± 13.72	61.72 ± 14.48*
*Systolic LV function*			
LVEF (%)	59.92 ± 5.65	58.44 ± 5.32	57.06 ± 3.29
LVGRS (%)	41.41 ± 5.14	39.18 ± 5.58	38.80 ± 6.24
LVGCS (%)	− 22.87 ± 4.11	− 19.05 ± 3.27*	− 18.27 ± 2.14*
LVGLS (%)	− 22.89 ± 3.05	− 18.41 ± 3.04*	− 16.72 ± 3.87*†
*Diastolic LV function*			
LVRSR (1/s)	− 2.31 ± 0.45	− 2.20 ± 0.35	− 2.05 ± 0.36
LVCSR (1/s)	1.36 ± 0.27	0.99 ± 0.26*	0.81 ± 0.24*†
LVLSR (1/s)	1.28 ± 0.26	1.10 ± 0.21*	0.94 ± 0.20*†
*RV structure*			
RVEDVI (mL/m^2^)	82.17 ± 13.32	80.69 ± 28.82	73.06 ± 18.59
RVESVI (mL/m^2^)	37.38 ± 7.05	37.47 ± 14.05	34.42 ± 8.86
SVI	44.79 ± 7.33	43.22 ± 15.19	38.64 ± 10.07
RVMI (g/m^2^)	10.46 ± 2.47	11.41 ± 2.86	12.64 ± 2.74*
*Systolic RV function*			
RVEF (%)	54.57 ± 3.12	53.73 ± 2.97	53.07 ± 2.61
RVGRS (%)	40.50 ± 3.26	39.48 ± 3.83	38.36 ± 3.94
RVGCS (%)	− 20.03 ± 2.79	− 18.72 ± 2.17	− 18.60 ± 3.00*
RVGLS (%)	− 21.46 ± 2.74	− 18.50 ± 2.32*	− 17.09 ± 3.11*†
*Diastolic RV function*			
RVRSR (1/s)	− 2.65 ± 0.87	− 1.81 ± 0.45*	− 1.44 ± 0.68*
RVCSR (1/s)	1.19 ± 0.18	0.85 ± 0.23*	0.76 ± 0.26*
RVLSR (1/s)	1.14 ± 0.18	0.87 ± 0.13*	0.75 ± 0.14*†

*LV* left ventricular, *RV* right ventricular, *EDVI* end-diastolic volume index, *ESVI* end-systolic volume index, *SVI* stroke volume index, *MI* mass index, *EF* ejection fraction, *GRS* global peak systolic radial strain, *GCS* global peak systolic circumferential strain, *GLS* global peak systolic longitudinal strain, *RSR* peak diastolic radial strain rate, *CSR* peak diastolic circumferential strain rate, *LSR* peak diastolic longitudinal strain rate

*p value < 0.05 when compared with the controls group

^**†**^*p* value < 0.05 when compared with the patients with HbA1c < 7.0%

### Associations between HbA1c and LV and RV strain in patients with T2DM

Table 3 reported the relationship between LV strain and HbA1c in T2DM patients. Unadjusted univariable linear regression analysis demonstrated a statistically significant association between HbA1c and LVGCS [*β* (95%CI) = 0.32 (0.12,0.52), *p* = 0.002], LVGLS [*β* (95%CI) = 0.374 (0.18, 0.57), *p* < 0.001], LVRSR [*β* (95%CI) = 0.26 (− 0.05, 0.46), *p* = 0.014], LVCSR [*β* (95%CI) =   − 0.23 (− 0.43, 0.02), *p* = 0.029] and LVLSR [*β* (95%CI) =   − 0.40 (− 0.59,  − 0.20), *p* < 0.001]. Multivariable linear regression analysis depicted an independent correlation between HbA1c and LVGLS [*β* (95%CI) = 0.27 (0.03, 0.51, *p* = 0.030)] and LVLSR [*β* (95%CI) =   − 0.39 (− 0.67,  − 0.11), *p* = 0.007] after adjustment for age, sex, BMI, diabetic duration, smoking, family history of CAD, hypertension, dyslipidemia, hypoglycemic mediation, FBG, eGFR and LVMI. Table 4 reported the relationship between RV strain and HbA1c in T2DM patients. Univariable linear regression analysis demonstrated that HbA1c was significantly associated with RVGLS [*β* (95%CI) = 0.59 (0.42, 0.76), *p* < 0.001], RVRSR [*β* (95%CI) = 0.29 (0.09, 0.49), *p* = 0.005], RVCSR [*β* (95%CI) =   − 0.37 (− 0.56,  − 0.17), *p* < 0.001] and RVLSR [*β* (95%CI) =   − 0.45 (− 0.64,  − 0.27), *p* < 0.001]. Multivariable linear regression analysis revealed that HbA1c was independently correlated with RVGLS [*β* (95%CI) = 0.56 (0.33, 0.80), *p* < 0.001] and RVLSR [*β* (95%CI) = − 0.39 (− 0.65,  − 0.14), *p* = 0.003].

**Table 3:** Association between the LV strain and HbA1c in diabetic patients

	LVGRS		LVGCS		LVGLS		LVRSR		LVCSR		LVLSR	
	*β* (95% CI)	*p*	*β* (95% CI)	*p*	*β* (95% CI)	*p*	*β* (95% CI)	*p*	*β* (95% CI)	*p*	*β* (95% CI)	*p*
*Model 1*												
HbA1c	− 0.09 (− 0.30, 0.12)	0.396	0.32 (0.12, 0.52)	0.002	0.37 (0.18, 0.57)	< 0.001	0.26 (− 0.05, 0.46)	0.014	− 0.23 (− 0.43, 0.02)	0.029	− 0.40 (− 0.59, − 0.20)	< 0.001
*Model 2*												
HbA1c	− 0.29 (− 0.56, 0.01)	0.051	0.04 (− 0.22, 0.28)	0.774	0.27(0.03, 0.51)	0.030	0.25 (− 0.03, 0.52)	0.083	− 0.03 (− 0.30, 0.25)	0.858	− 0.39 (− 0.67, − 0.11)	0.007

*Model 1* unadjusted regression analysis

*Model 2* adjusted for age, sex, BMI, diabetic duration, smoking, family history of CAD, hypertension, dyslipidemia, hypoglycemic mediation, FBG, eGFR and LVMI

**Table 4:** Association between the RV strain and HbA1c in diabetic patients

	RVGRS		RVGCS		RVGLS		RVRSR		RVCSR		RVLSR	
	*β* (95% CI)	*p*	*β* (95% CI)	*p*	*β* (95% CI)	*p*	*β* (95% CI)	*p*	*β* (95% CI)	*p*	*β* (95% CI)	*p*
*Model 1*												
HbA1c	− 0.16 (− 0.37, 0.05)	0.124	0.09 (− 0.12, 0.30)	0.412	0.59 (0.42, 0.76)	< 0.001	0.29 (0.09, 0.49)	0.005	− 0.37 (− 0.56, − 0.17)	< 0.001	− 0.45 (− 0.64, − 0.27)	< 0.001
*Model 2*												
HbA1c	− 0.27 (− 0.59, 0.04)	0.083	0.08 (− 0.19, 0.35)	0.547	0.56 (0.33, 0.80)	< 0.001	0.26 (− 0.02, 0.53)	0.067	− 0.18 (− 0.41, 0.05)	0.128	− 0.39 (− 0.65, − 0.14)	0.003

*Model 1* unadjusted regression analysis

*Model 2* adjusted for age, sex, BMI, diabetic duration, smoking, family history of CAD, hypertension, dyslipidemia, hypoglycemic mediation, FBG, eGFR and RVMI

### Mediation analysis

All mediation analyses were performed after adjustment for age, sex, BMI, diabetes duration, smoking, family history of CAD, hypertension, dyslipidemia, FBG, triglycerides, total cholesterol, high-density lipoprotein, low-density lipoprotein, eGFR and hypoglycemic therapy. The difference in LVGLS among patients with different levels of HbA1c was not mediated by RVMI, RVEF and RVGLS. The difference in LVLSR among patients with different levels of HbA1c was not mediated by RVMI, RVEF and RVLSR. Additionally, LVMI, LVEF and LVGLS had no significant mediating effects on RVGLS, and LVMI, LVEF and LVGLS showed no significant mediating effects on RVLSR (Additional file 1: Table S1).

### Intra-observer and inter-observer reproducibility of LV and RV strain parameters

The intra-observer and inter-observer reproducibility of LV and RV strain and strain rate were considered good (all ICCs > 0.75). The intra-observer and inter-observer correlation coefficients are represented in Table 5. The reproducibility of LV strain parameters was excellent. The ICC values in the intra-observer analysis were 0.892 (95% CI 0.798–0.931), 0.923 (95% CI 0.831–0.964), 0.919 (95% CI 0.825–0.960), 0.907 (95% CI 0.793–0.946), 0.931 (95% CI 0.867–0.972) and 0.925 (95% CI 0.0.866–0.981) for LVGRS, LVGCS, LVGLS, LVRSR, LVCSR and LVLSR, respectively. The ICC values in the inter-observer analysis were 0.854 (95% CI 0.786–0.879), 0.901 (95% CI 0.807–0.952), 0.896 (95% CI 0.814–0.943), 0.864 (0.761–0.927), 0.907 (95% CI 0.802–0.952) and 0.899 (95% CI 0.796–0.956) for LVGRS, LVGCS, LVGLS, LVRSR, LVCSR and LVLSR, respectively. The reproducibility of RV strain parameters was good to excellent. The ICC values in the intra-observer analysis were 0.783 (95% CI 0.707–0.852), 0.875 (95% CI 0.757–0.936), 0.894 (95% CI 0.797–0.941), 0.797 (95% CI 0.713–0.874), 0.881 (95% CI 0.758–0.943), 0.903 (95% CI 0.804–0.951) for RVGRS, RVGCS, RVGLS, RVRSR, RVCSR and RVLSR, respectively. The ICC values in the inter-observer analysis were 0.751 (95% CI 0.691–0.836), 0.838 (95% CI 0.755–0.873), 0.865 (95% CI 0.759–0.916), 0.762 (95% CI 0.706–0.855), 0.869 (95% CI 0.773–0.919) and 0.886 (95% CI 0.779–0.937) for RVGRS, RVGCS, RVGLS, RVRSR, RVCSR and RVLSR, respectively.

**Table 5:** intra-observer and inter-observer reproducibility of LV and RV strain and strain rate

	Intraclass correlation coefficient (95% CI)	
	Intra-observer	Inter-observer
LVGRS (%)	0.892 (0.798–0.931)	0.854 (0.786–0.879)
LVGCS (%)	0.923 (0.831–0.964)	0.901 (0.807–0.952)
LVGLS (%)	0.919 (0.825–0.960)	0.896 (0.814–0.943)
LVRSR (1/s)	0.907 (0.793–0.946)	0.864 (0.761–0.927)
LVCSR (1/s)	0.931 (0.867–0.972)	0.907 (0.802–0.952)
LVLSR (1/s)	0.925 (0.866–0.981)	0.899 (0.796–0.956)
RVGRS (%)	0.783 (0.707–0.852)	0.751 (0.691–0.836)
RVGCS (%)	0.875 (0.757–0.936)	0.838 (0.755–0.873)

## Discussion

This study showed that the occurrence of decreased biventricular strain in T2DM patients with preserved EF compared with controls, and suboptimal glycemic control (HbA1c ≥ 7%) aggravated the deterioration of biventricular strain in T2DM patients. HbA1c was independently associated with LVGLS, LVLSR, RVGLS and RVLSR after adjustment for risk factors in T2DM patients. Additionally, the relationships between HbA1c and LV (RV) strain were not statistically mediated by parameters of the other ventricular structure, function and strain in this diabetic cohort.

The prevalence of heart failure in T2DM patients is 22% [27] and LV diastolic dysfunction is the initial manifestation of diabetic cardiac pathology [4, 28, 29]. Increasing investigations found the occurrence of subclinical LV systolic and diastolic dysfunction in T2DM patients and considered that the EF is not a good indicator of early LV functional impairment [7, 22]. CMR tissue tracking could sensitively and reliably detect the abnormalities of both systolic and diastolic function in T2DM [22, 30]. The underlying mechanism of myocardial dysfunction in T2DM is complicated and multicomponent. Recently, some studies considered that a chronic increase in blood glucose levels could impair LV function via various mechanisms, such as modification in mitochondrial energy metabolism, elevation in myocardial oxidative stress, activation of the endothelin system and the renin–angiotensin–aldosterone system (RAAS), and the production of advanced glycosylation end products (AGEs) resulting in the damage of cardiomyocytes and imbalances of calcium homeostasis [15, 16, 31, 32]. Previous studies revealed the adverse effects of hyperglycemia on LV systolic longitudinal and circumferential strain in asymptomatic T2DM and detrimental subclinical decline in LV circumferential strain in obese adolescents with dysglycemia, indicating the importance of blood glucose control [33, 34]. Thus, reasonable and standard glycemic control may help alleviate myocardial injury and subsequent potential cardiovascular events. The formation of HbA1c is slow, continuous and irreversible, depending on the ambient glucose concentration. HbA1c could reflect glycemic status in the past 8–12 weeks and has been proven to superiorly estimate mean glycemia than conventional measurements of blood glucose concentration [35–39]. Studies showed that an approximate 1% decline in HbA1c is coupled with a 16% decreased exposure to heart failure, and poor glycemic control at admission was associated with all-cause mortality and major adverse cardiovascular and cerebrovascular events [40, 41]. However, results from large randomized trials disputed the merits of intensive glycemic control and the concept that strengthening blood glucose control with uniformity should be substituted by individualized HbA1c goals as an ideal target is increasingly recognized [42, 43].

The present study found that T2DM patients with suboptimal glycemic control (HbA1c ≥ 7.0%) had significantly lower systolic and diastolic longitudinal strain than those with HbA1c < 7.0%. These results indicated that improvements in glycemic control were likely accompanied by significant amelioration in LV function, consistent with previous studies [22, 44]. Longitudinal myocardial fibers were predominantly positioned in the sub-endocardium, and the wall layer was most vulnerable to microvascular ischemia. LV longitudinal strain and strain rate decrease were considered as the major and earliest markers of preclinical DM-related cardiac dysfunction [30, 45]. Although it is not clear whether the duration and degree of glycemic control impairment induce cardiac dysfunction, it supports the conception that HbA1c < 7% may be important for cardiac health [46]. A recent study demonstrated that poor blood glucose control was an independent predictor of all directions of peak systolic strain and peak diastolic strain rate in Chinese T2DM patients with normal LVEF [22]. However, our study only revealed a significant association between glycemic control and LV subclinical systolic and diastolic dysfunction in the longitudinal direction instead of all directions, which is independent of risk factors in T2DM patients with preserved EF. One of the possible explanations for this discrepancy was the duration of diabetes in the study population, considering that the duration of diabetes in the present study was less than in Zhou et al.’ study, and the HbA1c level was lower in the present study, indicating that our study population was at an earlier stage of diabetes and strain analysis could sensitively detect early myocardial damage relative conventional parameters. Therefore, focusing on alterations in LV strain may facilitate the clinical management of T2DM patients with poor glycemic control to reduce cardiovascular disease risk and improve outcomes.

Recently, the emphasis on RV functional evaluation has attracted substantial attention and RV dysfunction was considered to be associated with adverse outcomes [27, 47]. Although some studies reported the association between RV dysfunction and diabetes [8], information on the role of HbA1c in RV function was scarce, and the topic remained largely unexplored, probably due to certain technical limitations. RV functional evaluation was generally challenging due to sophisticated geometry and motion. CMR is the gold standard for assessment of RV structure and function and CMR tissue tracking strain analysis could provide a more comprehensive and accurate assessment of RV global and regional function. Therefore, the present study further extended previous research and applied CMR tissue tracking to explore the effects of glycemic control on RV function in T2DM patients. Parallel to the findings in the LV, the results demonstrated that RV longitudinal strain and strain rate were significantly lower in patients with suboptimal glycemic control than optimal glycemic control although RVEF and structure were unaffected. Furthermore, glycemic control was significantly associated with RV longitudinal systolic and diastolic dysfunction in T2DM patients. A recent study also delineated the significant relationship between RV longitudinal strain and HbA1c [48]. One of the possible underlying mechanisms for this finding may be poor glycemic control and subsequent hyperglycemia and the formation of advanced glycation, which could exert detrimental effects on myocardial calcium handling, leading to RV contractile and relaxed impairment [49]. These findings probably emphasized the underlying mechanistic role of glycemic control in RV dysfunction, as well as the fact that poor glycemic control may indicate subclinical myocardial dysfunction in patients with T2DM.

The present study showed that the associations of HbA1c with LV and RV strain were not statistically mediated by other ventricular structure and function changes after adjustment for risk factors, suggesting that blood glucose control may produce direct effects on the myocardium that interacted independently with each other in both ventricles. Even though a parallel impact may exist on LV and RV function in diabetes, some comparable pathophysiological pathways may have divergent influences on RV and LV structure and function in diabetes, possibly in part due to the differences in LV and RV anatomy, compliance, and pressure [19, 20]. Further investigation is necessary to determine whether improving blood glucose control can ameliorate biventricular dysfunction.

## Limitation

This research has several limitations. First, this is a retrospective single-center study with a relatively small sample size. Thus, our findings need to be further validated in multicenter studies with a larger population. Second, the cross-sectional design of the study cannot elucidate strong cause-and-effect associations. Third, the patients included in this study have a short diabetic duration, meaning that our findings may not necessarily be applicable to a wide range of diabetic patients, especially those with long-term diabetes. Fourth, clinical outcomes such as heart failure were not considered and evaluated in this study.

## Conclusion

The present study revealed that plasma glucose control was associated with the LV and RV systolic and diastolic function in T2DM patients with normal LVEF, independently of other traditional cardiovascular risk factors. This correlation was not statistically mediated by other ventricular changes, suggesting that HbA1c affected both LV and RV function probably via direct myocardial involvement.

## Supplementary Information

**Additional file 1: Table S1.** Mediation analyses: cardiac magnetic resonance biventricular strain analysis in diabetic patients. All analyses were adjusted for age, sex, BMI, diabetic duration, smoking, family history of CAD, hypertension, dyslipidemia, fasting blood glucose, triglycerides, total cholesterol, high-density lipoprotein, low-density lipoprotein, eGFR and hypoglycemic therapy.

## Data Availability

The datasets used and analyzed during the current study are available from the corresponding author on reasonable request.

## Abbreviations

BMI: Body mass index
CSR: Peak diastolic circumferential strain rate
EDVI: End-diastolic volume index
EF: Ejection fraction
eGFR: Estimated glomerular filtration rate
ESVI: End-systolic volume index
FBG: Fasting blood glucose
GCS: Global peak systolic circumferential strain
GLS: Global peak systolic longitudinal strain
GRS: Global peak systolic radial strain
HbA1c: Glycosylated hemoglobin
HDL: High-density lipoprotein
LDL: Low-density lipoprotein
LSR: Peak diastolic longitudinal strain rate
LV: Left ventricular
MI: Mass index
RSR: Peak diastolic radial strain rate
RV: Right ventricular
SVI: Stroke volume index
T2DM: Type 2 diabetes mellitus
