# Supplementary material for: Impact of glycemic control on biventricular function in patients with type 2 diabetes mellitus: a cardiac magnetic resonance tissue tracking study
Source: Insights Imaging. 2023 Jan 11;14:7. doi: 10.1186/s13244-022-01357-7 (PMC9833026; doi:10.1186/s13244-022-01357-7)
Supplement: Supplementary file 1 — Additional file 1: Table S1. Mediation analyses: cardiac magnetic resonance biventricular strain analysis in diabetic patients. All analyses were adjusted for age, sex, BMI, diabetic duration, smoking, family history of CAD, hypertension, dyslipidemia, fasting blood glucose, triglycerides, total cholesterol, high-density lipoprotein, low-density lipoprotein, eGFR and hypoglycemic therapy. [file 13244_2022_1357_MOESM1_ESM.pdf]

ELECTRONIC SUPPLEMENTARY MATERIAL

Impact of glycemic control on biventricular function in patients with type 2 diabetes mellitus: a cardiac magnetic resonance tissue tracking study

Additional table Mediation analyses: cardiac magnetic resonance biventricular strain analysis in diabetic patients

A: Associations between independent variable and mediators					
LVGLS	Β(95%CI)	P	RVGLS	β(95%CI)	P
- HbA1c-RVEF	-0.08 (-0.33, 0.17)	0.519	- HbA1c-LVEF	-0.10 (-0.36, 0.17)	0.459
- HbA1c-RVMI	-0.14 (-0.40, 0.13)	0.298	- HbA1c-LVMI	0.24 (-0.03, 0.51)	0.075
- HbA1c-RVGLS	-0.58 (-0.81, -0.35)	<0.001	- HbA1c-LVGLS	-0.23 (-0.47, -0.03)	0.031
LVLSR			RVLSR		
- HbA1c-RVEF	-0.08 (-0.33, 0.17)	0.519	- HbA1c-LVEF	-0.10 (-0.36, 0.16)	0.459
- HbA1c-RVMI	-0.14 (-0.40, 0.13)	0.298	- HbA1c-LVMI	-0.24 (-0.51, 0.03)	0.075
- HbA1c-RVLSR	-0.41 (-0.66, -0.16)	0.002	- HbA1c-LVLSR	-0.36 (-0.63, -0.08)	0.012
B: Associations between mediators and dependent variables					
	β(95%CI)	P		β(95%CI)	P
- RVEF-LVGLS	0.20 (-0.03, 0.54)	0.063	- LVEF-RVGLS	0.10 (-0.11, 0.30)	0.363
- RVMI- LVGLS	-0.15 (-0.05, 0.36)	0.140	- LVMI- RVGLS	-0.05 (-0.25, 0.15)	0.651
- RVGLS- LVGLS	0.10 (-0.14, 0.34)	0.405	- LVGLS- RVGLS	0.10 (-0.13, 0.32)	0.405
- RVEF-LVLSR	0.18 (-0.07, 0.44)	0.156	- LVEF-RVLSR	0.11 (-0.11, 0.33)	0.322
- RVMI- LVGLSR	-0.14 (-0.38, 0.11)	0.271	- LVMI- RVGLSR	-0.01 (-0.23, 0.21)	0.989
- RVLSR- LVLSR	0.17 (-0.08, 0.42)	0.187	- LVLSR- RVLSR	0.14 (-0.07, 0.36)	0.187
C: Associations between independent and dependent variables					

	β(95%CI)	P		β(95%CI)	P	
- HbA1c -LVGLS total	-0.23 (-0.01, -0.47)	0.048	- HbA1c -RVGLS total	-0.58 (-0.81, -0.35)	<0.001	
- HbA1c - LVGLS direct	-0.21 (-0.45, -0.02)	0.030	- HbA1c - RVGLS direct	-0.57 (-0.80, -0.34)	<0.001	-
- HbA1c -LVLSR total	-0.38 (-0.63, -0.08)	0.012	- HbA1c -RVLSR total	-0.41 (-0.66, -0.16)	0.002	
- HbA1c – LVLSR direct	-0.34 (-0.62, -0.06)	0.017	- HbA1c – RVLSR direct	-0.40 (-0.65, -0.14)	0.003	
D: Mediating effects						
	Δβ	CI		Δβ	CI	
- HbA1c -total- LVGLS	-0.02	(-0.09, 0.03)	- HbA1c-total -RVGLS	-0.01	(-0.06, 0.02)	
- HbA1c – RVEF-LVGLS	0.01	(-0.05, 0.09)	- HbA1c - RVEF-RVGLS	0.01	(-0.02, 0.06)	
- HbA1c -RVMI-LVGLS	-0.04	(-0.09, 0.02)	- HbA1c -RVMI-RVGLS	-0.03	(-0.09, 0.01)	
- HbA1c -RVGLS-LVGLS	0.01	(-0.03, 0.06)	- HbA1c -LVGLS-RVGLS	0.01	(-0.03, 0.05)	
- HbA1c -total- LVLSR	-0.04	(-0.08, 0.02)	- HbA1c-total -RVLSR	-0.01	(-0.07, 0.03)	
- HbA1c - RVEF-LVLSR	0.01	(-0.04, 0.07)	- HbA1c - LVEF-RVLSR	0.01	(-0.02, 0.04)	
- HbA1c -RVMI-LVLSR	-0.06	(-0.20, 0.05)	- HbA1c -LVMI-RVLSR	-0.04	(-0.08, 0.01)	
- HbA1c -RVLSR-LVLSR	0.01	(-0.02, 0.05)	- HbA1c -LVLSR-RVLSR	0.02	(-0.10, 0.15)	

All analyses were adjusted for age, sex, BMI, diabetic duration, smoking, family history of CAD, hypertension, dyslipidemia, fasting blood glucose, triglycerides, total cholesterol, high-density lipoprotein, low-density lipoprotein, eGFR and hypoglycemic therapy.
